# Percutaneous ultrasound-guided ablation of BW7756-hepatoma using ethanol or acetic acid in a rat model

**DOI:** 10.1186/1471-230X-7-45

**Published:** 2007-12-13

**Authors:** Enrico M Zardi, Domenico Borzomati, Fabio Cacciapaglia, Antonio Picardi, Sergio Valeri, Antonella Bianchi, Tommaso Galeotti, Giusy Coppolino, Roberto Coppola, Antonella Afeltra

**Affiliations:** 1 Department of Clinical Medicine "Campus Bio-Medico" University, Rome, Italy; 2 Department of Digestive Disease "Campus Bio-Medico" University, Rome, Italy; 3 Department of Pathology "Campus Bio-Medico" University, Rome, Italy; 4 Institute of General Pathology, Catholic University Medical School, Rome, Italy

## Abstract

**Background:**

To compare tumor necrosis in hepatoma induced in rats by a single percutaneous injection of ethanol (PEI) or acetic acid (PAI).

**Methods:**

BW7756 hepatomas of 1 mm^3 ^were implanted in the liver of 40 male healthy rats. After 14 days, the 36 surviving rats were treated, in a single session, by ultrasound-guided injection of 300 μl of 95% ethanol (n = 17) or 100 μl of 50% acetic acid (n = 19). They were sacrificed 14 days after treatment and explanted tumoral livers were examined. The same PAI procedure was repeated on 13 additional rats to exclude a suspected occurrence of technical failures during the experiment, due to a surprisingly high rate of deaths within 30 minutes after PAI.

**Results:**

Four rats died within four days after tumor implantation; after PEI, 1/17 (6%) died, whereas after PAI 9/19 (47%) died. The remaining 26 rats, after 14 days post-percutaneous ablation, were sacrificed. Gross and microscopic examinations showed that the hepatoma's nodules treated with PEI had 45.3 ± 19.4% tumor necrosis compared to 49 ± 23.3% (P = NS) for those treated with PAI. Complete tumor necrosis was not found in any animal. Peritoneal invasion was present in 4/16 (25%) and 2/10 (20%) rats treated with PEI or PAI, respectively (P = NS). Autopsy was performed in the 5 additional rats that died within 30 minutes after PAI.

**Conclusion:**

Our results show that there is no significant difference in the percentage of tumor necrosis between two local ablation methods in spite of the different dosages used. However, mortality in the PAI-treated group was greater than in PEI-treated group, presumably due to greater acetic acid systemic diffusion and its metabolic side effects. In human subjects, HCC occurs in the setting of cirrhosis, where the non-tumoral tissue is firmer than the tumor structure, with consequent reduction of drug diffusion. This could be the reason why some human studies have concluded similar or even better safety and efficacy with PAI compared to PEI.

## Background

Several methods of percutaneous ablation of hepatocellular carcinoma (HCC) have been developed worldwide, but which of these approaches is the most effective against this liver tumor is still an issue of debate [1,2].

Percutaneous acetic acid injection (PAI) has been reported to have a stronger cytotoxic effect and a lower rate of local recurrence than percutaneous ethanol injection (PEI) in the treatment of small HCCs [3,4]. The same authors underline that the necrosis capacity of PAI is equivalent to that of PEI at an acetic acid concentration of 15% whereas a greater killing effect is reached at a 50%. Other authors have also shown that PAI is effective in the treatment of small HCCs in a single high-dose session [5].

In humans, PEI is known to be the best and most common treatment for HCCs smaller than 3 cm when there are no more than three tumoral lesions, resulting in induction of necrosis as a result of cellular dehydration, protein denaturation, and chemical occlusion of tumor vessels [6]. However, some authors believe that PEI necessitates more treatment sessions than PAI and has a higher rate of local recurrence [4].

The aim of this study was to evaluate safety and efficacy of a single ultrasound-guided session of PEI in comparison to a single session of PAI in male rats with BW7756 hepatoma [7-9].

## Methods

Approval for this protocol was obtained from the Ethical Committee of the Catholic University of Rome.

### Animals

Fifty-three male healthy rats (weight 250–260 g, age 14 weeks), housed in autoclaved cages under barrier-sustained conditions with controlled temperature (27°C) and humidity (30%), were used.

All animal studies were performed in accordance with the regulations of the National Institute of Health.

### Tumor implantation

BW7756 murine hepatoma, an extensively studied tumor that undergoes an exponential growth spurt in the 14–21 days post-implantation, was obtained from the Institute of General Pathology (Catholic University of Rome).

Before starting the implantation procedure, anaesthesia was induced in all rats by an intramuscular injection of a mixture of 2 ml of Ketamine and 1 ml of Medetomidine.

Open laparotomy was performed through midline xifo-pubic incision to expose the liver by means of mosquito forceps and 1 mm^3 ^of BW7756 hepatoma was directly inserted into the liver of all rats following a modified Yang technique [10]. Intrahepatic tumor implantation was carried out after a small incision was done in the liver for the purpose of both hemostasis and formation of a tension-free pocket to accept the hepatoma; to this site the tumor was secured with resorbable microsuture. After the implantation, the liver with the fastened tumor was reintroduced into the abdominal cavity and the abdomen was closed using resorbable sutures. Post-operatively, the animals were evaluated daily for diarrhea, loss of hair, food intake and unusual behaviour.

### PEI and PAI procedures

After 14 days post-hepatoma implantation, the liver of all rats was sonographically revaluated to check the tumor volume and percutaneous ultrasound-guided ablation was performed immediately, using General Electric 400 equipment and a convex 10 MHz probe. Rats were randomized before starting the procedure and then were anesthetized anew as previously described. They were placed in the supine position, the abdominal area was accurately shaved and disinfected, and all 36 rats were treated, in a single session: 17 with percutaneous (300 μl 95%) ethanol injection (PEI) and 19 with (100 μl 50%) acetic acid injection (PAI). The different dosage was decided considering different tissue distribution and greater diffusion of acetic acid compared to ethanol [11].

In addition, to exclude that technical failures were the cause of the observed high number of deaths among the PAI treated rats, we repeated the experiment with the same modalities treating another consecutive number of rats (13 rats) with acetic acid injection (100 μl 50%).

### Pathologic examination

After 14 days from percutaneous injection, all rats (age of 18 weeks) were sacrificed by cervical dislocation and the liver was explanted en bloc and fixed in 10% buffered formaldehyde. At the end of the second experiment with PAI, autopsy was performed on the five rats that died within 30 minutes after the procedure.

After gross observation of the liver and the abdominal cavity, tissue samples were embedded in paraffin and then processed conventionally.

Five micron tissue sections were stained with hematoxylin-eosin (H&E) for microscopic examination at magnifications between 10× and 40× using Nikon ECLIPSE E1000M microscopy.

The necrosis percentage is expressed as the mean ratio between the necrotic area and the total tumor area (×100) of ten fields, observed through panoramic lens.

### Statistical analysis

All data are expressed as the mean ± SD. Data were analysed using the Prism statistical package (Graphpad Instat, version 3). Throughout this study, Fisher's exact test and Mann-Whitney's test for two-tail nonparametric variables were used to determine if there were differences in the studied groups. The results with a P-value < 0.05 were considered significant.

## Results

Within one week after BW7756 hepatoma implantation, 4/40 rats (10%) died from post-surgical complications and tumor growth that locally invaded the surrounding organs, causing ascites and metastases to the lung.

The 36 surviving rats were randomly divided into two groups: 17/36 (47.2%) for PEI and 19/36 (52.8%) for PAI ablation. Before percutaneous injection, the hepatomas mean volumes were: 1217 ± 300 mm^3 ^and 1320 ± 100 mm^3 ^in the PEI and PAI treatment groups, respectively (P=NS), and no extrahepatic infiltration was detected.

Subsequently, all rats underwent ultrasound-guided percutaneous injection of the neoplastic nodules.

**Figure 1:**
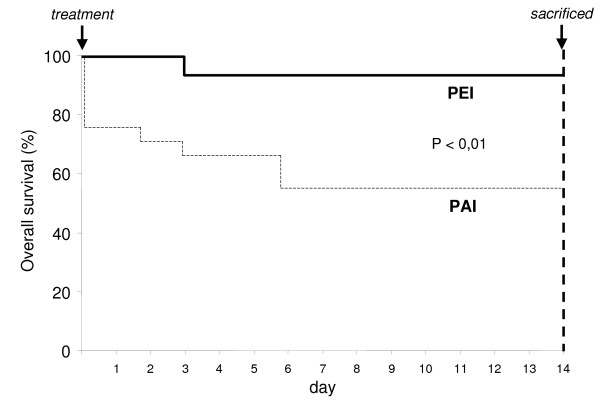
Comparison of overall survival between rats with hepatoma, treated with percutaneous ethanol injection (PEI) and percutaneous acetic acid injection (PAI), before being sacrificed.

Among PEI treated rats, one out of 17 (6%) died three days after the procedure, while in the PAI group 9/19 rats (47%) died within one week after the procedure (P < 0.01) (Figure 1). Pathologic examination disclosed a peritoneal and vessel neoplastic infiltration as the cause of death in the PEI-treated rat and in four out of nine PAI-treated rats (P=NS). Autopsy was not performed in the other five PAI-treated rats died within 30 minutes after the procedure.

After 14 days post-percutaneous ablation, all surviving rats were sacrificed: gross and microscopic examinations showed that peritoneal invasion was present in 4/16 (25%) and 2/10 (20%) rats receiving PEI and PAI treatment, respectively (P = NS), whereas in no lesion was complete necrosis found.

**Figure 2:**
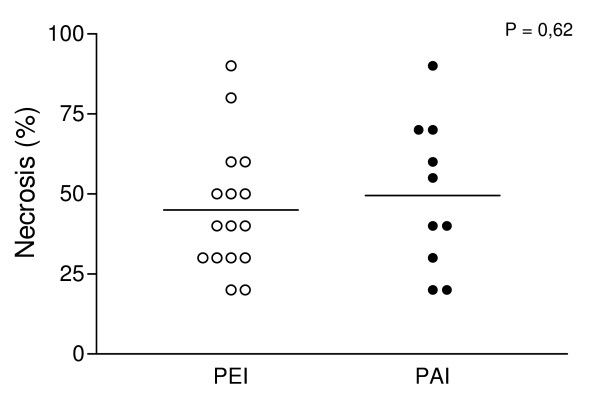
Comparison of necrosis percentage induced by percutaneous acetic acid injection (PAI) and percutaneous ethanol injection (PEI).

**Figure 3:**
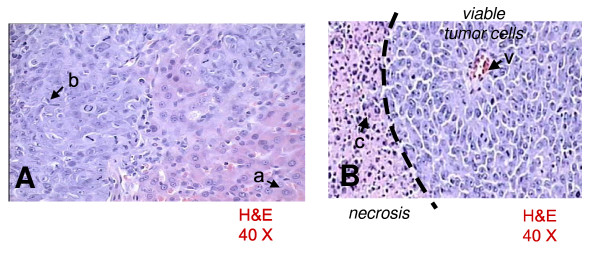
[A] Normal hepatocytes (a) and hepatoma cells (b). [B] Over 60% of field is occupied by perivascular (v) viable tumor cells; the left side is occupied by necrosis with cellular debris (c).

**Figure 4:**
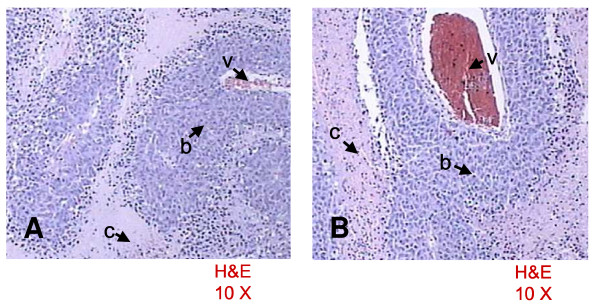
Representative histologic specimens of the liver after PEI [A] and PAI [B]. Perivascular (v) viable tumor cells (b) and necrosis with cellular debris (c).

The calculated percentage of necrosis produced with the injection of 300 μl of 95% ethanol was 45.3 ± 19.4%, whereas with the injection of 100 μl of 50% acetic acid was 49 ± 23.3% (P = NS) (Figure 2). There were no differences evident in the necrosis determined by acetic acid or ethanol. Histological changes after injection of either agent determined areas of reduced cell number with degeneration of cytoplasmic and nuclear components and cellular debris (Figure 3), intermingled with viable neoplastic cells localized in perivascular and central areas of the hepatoma (Figure 4).

In this first experiment we observed a higher number of deaths in the PAI treated rat group compared to the PEI treated rat group, which seemed somewhat surprising to us. Therefore, we repeated the PAI procedure treating a consecutive number of 13 rats with 100 μl of 50% acetic acid injection to exclude technical failures; we obtained the same number of dead rats (n. 5) within 30 minutes after the procedure as observed in the previous experiment. Autopsy was promptly carried out on these 5 rats (5/13 – 38.5%) and pathologic examination showed that the cause of death was a massive (about 40%) liver necrosis with diaphragma involvement observed in three rats or a complete inferior *vena cava *thrombosis with extension to right atrium (probably as a result of the activation of blood coagulation factors) seen in the remaining two rats.

The calculated percentage of necrosis produced by the injection of acetic acid in the eight surviving rats of this new experiment was 54 ± 28.3% (the difference with that of the previously studied rats is not statistically significant).

## Discussion

Acetic acid injection and ethanol injection are considered to be a rational alternative to surgical resection and, in particular, PEI seems to be the safest and most effective percutaneous treatment for resectable and unresectable HCCs <3 cm in humans [6,12-14]. Several studies have emphasized the suitability of both PEI and PAI for multiple session treatment of human HCCs [3,6,13-16].

Single high doses of PEI and PAI have also been investigated to evaluate safety and efficacy in human HCCs with good quality results [5,14,17].

An interesting study performed in patients with HCC on the waiting list for liver transplantation showed that most patients with solitary nodules <4 cm who received PEI had 90% to 100% tumor necrosis [18].

However, some studies have compared the effectiveness of PEI and PAI in HCCs of humans or in liver tumor of animals showing a greater efficacy of PAI compared to PEI [1,4,19,20]; on the other hand, human studies only compared local recurrence and survival rates without evaluating tumor necrosis whereas, to our knowledge, the only study carried out on animals evaluated just the area of coagulation after 30 minutes from the injection of ethanol or acetic acid in the liver tumor [20].

An interesting previous study in healthy rats compared the effect of the injection of 300 μl of pure ethanol and 100 μl of 50% acetic acid separately into different sites of the same liver. Acetic acid showed a more homogeneous distribution and better infiltrating ability than pure ethanol [11].

Basing on this experiment, we evaluated in our study safety, efficacy and percentage of necrosis produced in a liver tumor with a single ultrasound-guided session of PEI (300 μl 95%) in comparison to an equal single session of PAI (100 μl 50%) in male rats with BW7756 hepatoma, sacrificed 14 days after the procedure.

We chose to compare PAI and PEI at different dosages since it is already known that, volume being equal, acetic acid is able to induce an area of necrosis of hepatic tumors three times greater than that caused by ethanol according to some authors, and even ten times greater according to others. In fact, a previous study showed that acetic acid produced significantly larger zones of tumor coagulation compared with ethanol when injected in equal volumes into VX2 carcinoma in rabbits killed 30 minutes after the procedure [20].

Conversely, information is lacking about the safety of both treatments in rats with hepatoma sacrificed 14 days after the procedure.

The choice of comparing PAI and PEI at different dosages (100 μl for PAI and 300 μl for PEI) also depends on the fact that acetic acid has a greater diffusion through tumoral tissue than ethanol, and, therefore, a smaller quantity is needed to kill tumor cells [20]. On the other hand, increasing the dosage of acetic acid to values equivalent to those of ethanol (300 μl) probably caused more deaths among rats treated with PAI while decreasing the dosage of the well-tolerated ethanol to values equivalent to those for acetic acid (100 μl) might have been inadequate for PEI treatment. Studies on safety of these methods have shown that risk and complications rates of PAI treatment are nearly superimposable to those of PEI [21-23].

On the contrary, our results show no significant difference between PEI and PAI as to the efficacy (percentage of necrosis and histological changes), but significant difference as to safety (6% of deaths after PEI vs. 47% after PAI). These data were confirmed by repeating the experiment with PAI procedure in 13 rats and performing an autopsy in the 5 rats died within 30 minutes after the procedure, thus demonstrating that the high number of deaths (5/13 rats) was not due to technical failure but to acute complications.

However, this may not automatically apply to humans as the majority of hepatocellular carcinomas in human subjects occur in the setting of cirrhosis where the non-tumoral tissue is firmer than the tumor structure and acetic acid, as well as ethanol, cannot diffuse much in the surrounding non-tumoral tissue. This could be the reason why some human studies have concluded similar or even better safety and efficacy when comparing PAI to PEI [1,4,5,19]. A recent study in patients awaiting liver transplantation showed that PEI induced complete necrosis of HCC in 65% of cases, whereas in patients with HCC undergoing PAI treatment, complete response was obtained in 69% of cases [24,25]; however, doubt arises about the real value of this complete response after PAI treatment because of the lack of histological evidence.

## Conclusion

In our study on animals, we did not observe complete necrosis either with PEI or PAI treatment, because the single treatment session we performed on each animal would not be sufficient to cause it.

Interestingly, a single dose of treatment gave a percentage of necrosis of nearly 50% for both percutaneous treatments, with a mortality rate quite lower for PEI than PAI. It is true that the same percentage of necrosis as PAI was obtained with a three times higher dosage of PEI, but in spite of this, PEI proved to be safer than PAI. Therefore, our data principally show that the ability of a single treatment session to cause tumor necrosis is nearly 50% both for PAI and PEI. However, in choosing the best possible chemical percutaneous treatment, both efficacy and adverse reactions have to be taken into consideration.

## Competing interests

All authors read and approved the final manuscript and declare that they have no competing interests.

## Authors' contributions

EMZ: participated in the design of the study, performed the ultrasonographic evaluation and percutaneous treatment, and drafted the manuscript;

DB: participated in the design of the study and carried out the surgical technique;

FC: participated in the design of the study, performed the statistical analysis and drafted the manuscript;

AP: participated in the design and coordination of the study, performed percutaneous treatment and helped to draft the manuscript;

SV: carried out the surgical technique;

AB: carried out the histological evaluation;

TG: carried out the histological evaluation;

GC: performed the bibliographic research and helped to draft the manuscript;

RC: conceived of the manuscript and participated in its design and coordination;

AA: conceived of the manuscript and participated in its design and coordination.

## Pre-publication history

The pre-publication history for this paper can be accessed here:


